# Induction of labour should be offered to all women at term: FOR: Induction of labour should be offered at term

**DOI:** 10.1111/1471-0528.15933

**Published:** 2019-10-03

**Authors:** Kate Lightly, Andrew D Weeks

**Affiliations:** ^1^ Sanyu Research Unit Liverpool Health Partners University of Liverpool and Liverpool Women’s Hospital Liverpool UK

There is now a large and growing body of literature that clearly shows that induction of labour at or beyond term is safer for mothers and babies, is well tolerated by patients, and has health economic benefits, when labour is well managed. There is an urgent need to translate these research findings into clinical practice and save the lives of more babies.

A large Cochrane review of 30 randomised trials (12 470 women) found that a policy of labour induction at or beyond term is associated with numerous benefits compared with expectant management: fewer perinatal deaths (risk ratio, RR 0.33), fewer stillbirths (RR 0.33), fewer babies with low Apgar scores (RR 0.70), and lower neonatal intensive care unit admission rates (RR 0.88). For the mother, there were fewer caesarean births (RR 0.92) but the rate of operative vaginal births was marginally increased (RR 1.07). There were no differences in other maternal outcomes, including perineal trauma and length of stay (Middleton et al. *Cochrane Database Syst Rev* 2018;5:CD004945).

These results are supported by the recent ARRIVE trial (Grobman et al. *N Engl J Med* 2018;379:513–23) in which 6106 nulliparous women at low risk were randomised to induction at 39 weeks of gestation or expectant management. Women who were induced had caesarean rates reduced from 22.2 to 18.6% and perinatal adverse outcomes reduced by 23%. The move to induction is also supported by economic analysis: a cost–utility analysis from the 35/39 trial found a mean cost saving of £263 per birth in the induction group (Walker et al. *BJOG* 2017;124:929–34).

Although research clearly shows the medical benefits of induction, it is argued that induction medicalises labour and provides a negative experience, and so must be unacceptable to women. This is not borne out by the evidence, however. A qualitative systematic review found that the anecdotal descriptions of ‘long, painful and risky’ are not echoed in qualitative research themes. Instead, the women interviewed reported that their birth priorities were ownership and an understanding of the process, control, social arrangements, relationships with staff, privacy, ‘enduring’ the hospital and keeping to established rhythms (Coates et al. *Midwifery* 2019;69:17–28).

The evidence shows that induction at term improves outcomes, reduces costs and improves a woman's sense of control. Therefore, it is our role as advocates for women to create system change and to reconfigure services to deliver more low‐risk inductions. Current induction pathways, which usually include inpatient consultant care with repeated cardiotocography, were set up to safely induce women at high risk, but the regimens can surely be modified for the induction of low‐risk births. One example is outpatient induction, which has been shown to be safe and acceptable, and is already routine care in Scandinavia. In Liverpool, women at low risk who labour following prostaglandin cervical ripening alone receive the benefits of midwifery‐led care.

Induction at term may not be for everyone, but it is a sensible and valid birth choice for all. In light of the evidence, it is certainly unethical to refuse a request for term induction and, in the era of Montgomery, could even raise legal concerns.

## Disclosure of interests

Andrew Weeks runs an information website, misoprostol.org, on a voluntary basis. He also acts as a scientific advisor to Azanta A/S. In this role he receives no personal remuneration other than travel expenses, but money is paid to the University of Liverpool for his time. Kate Lightly had no disclosures of interest to declare. Completed disclosure of interests form available to view online as supporting information.

## Contribution to authorship

AW conceptualised the article, KL performed the literature search and wrote the first draft of the article, AW made significant edits to the article and is the guarantor.
